# Hate speech detection with ADHAR: a multi-dialectal hate speech corpus in Arabic

**DOI:** 10.3389/frai.2024.1391472

**Published:** 2024-05-30

**Authors:** Anis Charfi, Mabrouka Besghaier, Raghda Akasheh, Andria Atalla, Wajdi Zaghouani

**Affiliations:** ^1^Information Systems Department, Carnegie Mellon University, Doha, Qatar; ^2^College of Humanities and Social Sciences, Hamad Bin Khalifa University, Doha, Qatar

**Keywords:** natural language processing, hate speech, Arabic language, dialectal Arabic, dataset annotation, Arabic corpora

## Abstract

Hate speech detection in Arabic poses a complex challenge due to the dialectal diversity across the Arab world. Most existing hate speech datasets for Arabic cover only one dialect or one hate speech category. They also lack balance across dialects, topics, and hate/non-hate classes. In this paper, we address this gap by presenting ADHAR—a comprehensive multi-dialect, multi-category hate speech corpus for Arabic. ADHAR contains 70,369 words and spans four language variants: Modern Standard Arabic (MSA), Egyptian, Levantine, Gulf and Maghrebi. It covers four key hate speech categories: nationality, religion, ethnicity, and race. A major contribution is that ADHAR is carefully curated to maintain balance across dialects, categories, and hate/non-hate classes to enable unbiased dataset evaluation. We describe the systematic data collection methodology, followed by a rigorous annotation process involving multiple annotators per dialect. Extensive qualitative and quantitative analyses demonstrate the quality and usefulness of ADHAR. Our experiments with various classical and deep learning models demonstrate that our dataset enables the development of robust hate speech classifiers for Arabic, achieving accuracy and F1-scores of up to 90% for hate speech detection and up to 92% for category detection. When trained with Arabert, we achieved an accuracy and F1-score of 94% for hate speech detection, as well as 95% for the category detection.

## 1 Introduction

Language, as a fundamental element of human expression, plays a pivotal role in shaping our interactions, beliefs, and perceptions, but it can also be a double-edged sword. In the digital age, where communication knows no bounds, the rise of hate speech has become a big concern. Hate speech is typically defined as any form of communication that belittles an individual or a group based on attributes such as race, color, ethnicity, gender or religion (Tontodimamma et al., 2021). It has become a serious issue, especially online, where people should be able to communicate freely and respectfully. Despite being conveyed through just a few words, hate speech produces an enduring and severe negative influence and its consequences go beyond the initial incident. It has the potential to produce social division, emotional distress, normalization of prejudice, and many other negative consequences (Bilewicz and Soral, 2020). Therefore, fighting this type of content became a necessity, to protect the society and the generations to come from being exposed to such harmful content.

In recent years, we witnessed a growing focus on developing resources and benchmarks to advance research on hate speech detection. Several resources exist for languages such as English. However, for Arabic, quality annotated hate speech datasets remain scarce. This hinders research and system development for automatic hate speech detection in Arabic. Unlike other languages, Arabic has a wide variety of dialects to the level that people in the Gulf or in Iraq hardly understand Maghrebi dialects such as Moroccan or Algerian. There is a pressing need to develop diverse Arabic hate speech corpora covering various dialects and topics to advance this research area. While some prior works created Arabic hate speech datasets, there are still gaps that need to be addressed. Several existing resources are specifically focused on individual dialects. For example, Haddad et al. (2019) introduced a corpus for the Tunisian dialect and Mulki et al. (2019) proposed a corpus specifically for the Levantine dialect. Some other corpora include Arabic text from mixed dialects such as Albadi et al. (2018), Omar et al. (2020), Duwairi et al. (2021), and Mubarak et al. (2022). However, they lack annotation specifying the dialect of each sentence and they also lack balancing between the different dialects. Hence, the dialect cannot be used as input to the hate speech detection task. In fact, knowing the dialect is crucial because Arabic dialects vary significantly, and some words can even have different meanings depending on the dialect. Certain words that may be considered normal or safe in one dialect could be perceived as offensive in another.

The use of Arabic dialects is prevalent in social media posts and this presents some additional challenges for natural language processing. Furthermore, existing datasets either do not cover any specific category of hate speech or cover just one category of hate speech like religion-related hate speech or gender-related hate speech. Another issue is that most existing datasets are imbalanced with respect to the distribution of hate speech vs non-hate speech. Often the hate speech is significantly less than the normal/safe content, which can bias machine learning models trained on them. Our work aims to address these limitations.

In this paper, we present a novel multi-dialectal and multi-category hate speech dataset that contains 70,369 words and 4,240 Arabic tweets. This dataset covers four language variants: MSA, Egyptian, Jordanian/Palestinian, Gulf, and Maghrebi. Additionally, we incorporated Modern Standard Arabic (MSA) as the main variant of Arabic, which is used in official communication. We collected the data from Twitter and each tweet was manually annotated by two annotators. The dataset is divided into two main classes, “Hate” and “Not Hate”. For each of the classes our dataset includes four sub-classes: nationality, religion, ethnicity, and race. We ensured a balanced distribution across dialects, hate/non-hate classes and the four sub-classes of nationality, religious beliefs, ethnicity, and race. We also report on several machine learning experiments that we conducted to evaluate the utility of our dataset for hate speech detection.

The remainder of this paper is organized as follows: Section 2 examines prior research on hate speech datasets and hate speech detection methods with special focus on Arabic. Section 3 presents our new hate speech dataset, detailing the methodologies for data collection and annotation. Section 4 reports on the machine learning experiments conducted on our corpus and their results. Section 5 discusses the strengths and limitations of our work. Section 6 summarizes our work, discusses its impact and outlines directions for future work.

## 2 Related work

Hate speech detection has been a popular task for the past few years. Several works focused on hate speech detection in different languages including English (Saleh et al., 2023), Italian (Polignano et al., 2019), Spanish (Plaza-del Arco et al., 2021), Indonesian (Marpaung et al., 2021), among others. The common approach across these studies involves using the BERT model for training their respective datasets.

One notable work (Mazari et al., 2023) presented a novel approach to multi-aspect hate speech detection by leveraging ensemble learning techniques that combined BERT with Deep Learning models like Bi-LSTMs and Bi-GRUs. These models were trained individually and then their outputs were combined to improve the precision of hate speech detection, particularly in identifying various forms of toxic language such as “identity hate”, “threat”, “insult”, “obscene”, “toxic”, and “severe toxic”. Evaluation metrics including ROC-AUC, Recall, Precision, F1-score, and MCC were used to assess model performance on the Kaggle hate speech comment classification challenge dataset in the English language. Results indicated that the ensemble-based models outperformed individual models, with the best proposed model achieving a high ROC-AUC score of 98.63% and demonstrating a reduction in misclassifications, thereby enhancing the precision of hate speech detection.

Another work by Caselli et al. (2021) introduced HateBERT, which is a specialized BERT model trained to detect abusive language in English using the RAL-E dataset sourced from banned Reddit comments. Their comparison with a generic pre-trained model across three English datasets (OffensEval 2019, AbusEval, and HatEval) consistently demonstrated HateBERT's superior performance in detecting offensive language, abusive language, and hate speech, specifically when it comes to the results of the macro-averaged F1 of the positive and negative classes.

On the other hand, there has been limited work when it comes to detecting hate speech in the Arabic language, and particularly when considering the diverse Arabic dialects. In the following, we provide an overview of the most recent papers on hate speech detection in Arabic as well as an in-depth discussion for the existing datasets as shown in Table 1.

**Table 1:** Available hate speech datasets for Arabic.

**References**	**Sources**	**Size**	**Annotation**	**Language variants**
Albadi et al. (2018)	Twitter	6,000 tweets	- Religious hate - Not hate	Arabic
Mulki et al. (2019)	Twitter (timeline of politicians, social/ political activists, and TV anchors)	5,846 political tweets	- Normal, - Abusive, - Hate	Syrian and Lebanese
Haddad et al. (2019)	Comments collected from different social media platforms	6,024 comment	- Normal, - Abusive, - Hate	Tunisian
Ousidhoum et al. (2019)	Twitter	13,000 tweets including 3,354 tweets in Arabic	- Abusive, - Hateful, - Offensive, - Disrespectful, - Fearful, - Normal	English, French, and Arabic
Omar et al. (2020)	Facebook, Twitter, YouTube, and Instagram	20,000 posts, tweets, and comments	- Hate, - Not hate	Standard Arabic
Duwairi et al. (2021)	9833 tweets	Twitter	-Hate, Abusive, or Normal, -Misogyny, Racism, Religious Discrimination, Abusive, or Normal	Mixed Arabic Dialects mainly Levantine
Mubarak et al. (2022)	Twitter	13,000 tweets (65% clean, 35% offensive)	-Clean, -Offensive: race, religion, ideology, disability, social class, gender, vulgar and violence	Mixed Arabic dialects (unspecified dialects)

In Magnossão de Paula et al. (2022), the authors developed transformer-based models like AraBERT and XLM-Roberta for detecting offensive language, hate speech, and fine-grained hate speech in Arabic tweets, finding ensemble methods achieved top results compared to individual models. In Almaliki et al. (2023), the authors developed the Arabic BERT-Mini Model (ABMM) to identify hate speech in Arabic Twitter content, categorizing texts into normal, abusive, or hate speech. However, their study did not provide specific statistics regarding the Arabic dialects represented in their dataset. Experimental results demonstrated that the ABMM achieved an impressive accuracy of 98.6% in detecting Arabic hate speech, surpassing the performance of earlier models. In Al-Ibrahim et al. (2023), the authors developed deep learning models like bidirectional LSTM and CNN for identifying hate speech in Arabic Twitter content, without focusing on any particular dialect. Outperforming traditional ML models; the top bidirectional LSTM model achieved 92.2% accuracy and 92% F1-score on an Arabic dataset of 15K tweets.

In Albadi et al. (2018), the authors presented a dataset for hate speech detection, which consists of 6,000 labeled and annotated tweets. This dataset was used for the task of detecting religious hate speech in Arabic social media. However, this study did not address any particular Arabic dialects. Additionally, the researchers introduced three Arabic lexicons containing religious hate terms, each accompanied by polarity and strength scores. They applied three approaches to detect religious hate speech, namely lexicon based, n-gram based, and deep learning-based approaches. The Recurrent Neural Network (RNN) architecture with Gated Recurrent Units (GRU) and pre-trained word embeddings gave the best performance with an accuracy of 0.79.

In Mulki et al. (2019), the authors introduced a Levantine Hate Speech and Abusive Twitter dataset called L-HSAB, which included 5,846 tweets divided into three categories: normal, abusive, and hateful. This dataset was created by crawling Twitter for tweets and manually labeling them by three annotators following a set of rules. L-HSAB was evaluated in machine learning-based classification experiments using Naive Bayes (NB) and Support Vector Machine (SVM). The experimental results demonstrated that for multi-class classification (i.e., Abusive, Hate, or Normal), the Naive Bayes classifier gave the best performance, achieving a precision of 86.3%, recall of 70.8%, F1-score of 74.4%, and an accuracy of 88.4%.

Another dataset was proposed by Haddad et al. (2019) under the name of T-HSAB, which includes 6,039 sentences from the Tunisian dialect and focusing mainly on social and political issues. This dataset was collected from different social media networks and the sentences were labeled as hate, abusive or normal. This dataset is unbalanced with a distribution of 63.49% normal sentences, 18.66% abusive sentences and 17.85% hate sentences. So, the hate speech and abusive content is around 36%. The authors conducted machine learning experiments on that dataset using two classifiers, namely SVM and Naive Bayes (NB). The best results were obtained when using NB with an accuracy of 92.9% and F1-score of 92.3%.

In Ousidhoum et al. (2019), the authors presented a multilingual multi-aspect hate speech analysis dataset, which was collected from Twitter and made out of three languages: English, Arabic and French. It included 5,647 English tweets, 4,014 French tweets, and 4,240 Arabic tweets. The authors used five attributes to classify the sentences: Directness, Hostility, Target, Group, and Annotator. Within these attributes, multiple labels were considered. For instance, “Directness” contained labels such as directness or indirectness, while “Hostility” included labels like abusive, hateful, and offensive. Similarly, “Target” comprised labels such as gender, origin, and religion, “Group” encompassed designations like individual and women, and “Annotator” included feelings such as disgust, anger, and shock. To annotate the data, native speakers were recruited from Amazon Mechanical Turk and they were selected based on their high reputation scores. The authors used several models to conduct the experiments such as Majority Label, LR (Linear Regression), STSL (Single Task Single Language), MTSL (Multitask Multilingual), STML (Single Task Multilingual), and MTML (Multitask Multilingual Models). Their results showed that deep learning methods outperformed classic BOW-based models in the majority of the multi-label classification problems. The best performance for Arabic was achieved by STSL, with a macro F1 score of 0.84 and a micro F1 score of 0.72.

In Mubarak et al. (2022), the authors introduced an Arabic multi-dialectal dataset which consists of 12,698 tweets classified into two main classes: clean and offensive. The offensive class was further classified into eight sub-classes which are: gender, race, ideology, social class, religion, disability, vulgar, and violence. While the dataset has a good balance between the main classes, “clean” and “offensive,” there is an imbalance among the sub-categories. The authors conducted experiments with various classical and pre-trained bert models. They obtained their best results with AraBERT with an accuracy of 92.64%, precision of 81.04%, recall of 79.31%, and an F1-score of 80.14%.

In Omar et al. (2020), the authors presented a multi-platform dataset for standard Arabic hate speech and abuse detection, which includes 20,000 sentences mainly in MSA retrieved from various social media platforms such as Twitter, Facebook, Instagram, and Youtube. The annotation was done manually using two labels: hate and not hate. The experiments on this dataset were conducted using 12 machine learning models, including MultinomialNB, SGD, Complement NB, LogisticRegression, Recurrent Neural Network(RNN), CNN and others. RNN outperformed the other classifiers with an accuracy and F1-score of 98.7%. A significant limitation of this work is that it categorizes both offensive and hate speech under the same label, annotating both types as hate speech.

ArHS created by Duwairi et al. (2021) is a hate speech data set for Arabic, which includes 9,833 tweets retrieved through the Twitter API. These tweets were categorized into five classes: misogyny, racism, religious discrimination, abusive language, and normal tweets. This dataset is quite imbalanced and does not consider the specific Arabic dialects used in the sentences. Among the tested models, the CNN classifier gave the best results with an accuracy rate and F1-score of 81%.

Two shared tasks were organized on the topic of hate speech detection for Arabic, particularly the shared tasks proposed by OSACT 4 and 5. The Shared Task proposed by OSACT 4^1^ comprised two sub-tasks: detecting offensive language (unacceptable or vulgar content) and identifying hate speech. Both sub-tasks used the same corpus, which includes 10,000 tweets manually annotated with labels indicating whether they contain offensive or non-offensive content, and if they involve hate speech or not. The corpus used in these shared tasks is very imbalanced, with only 5% of the content being annotated as hate speech (Mubarak et al., 2020). On the other hand, the shared task by OSACT 5^2^ comprised three sub-tasks namely, detection of offensive language, detection of hate speech and detection of the fine-grained type of hate speech. In this shared task, the used dataset is the one proposed by Mubarak et al. (2022), which has a good balance between the classes “clean” and “offensive” but is still imbalanced w.r.t. the sub-classes.

Even though multi-dialectal datasets were proposed by Duwairi et al. (2021) and Mubarak et al. (2022), these works did not take into account the distribution of instances across each dialect. It is possible that some dialects were collected more frequently than others, leading to an imbalance in the distribution of dialects in these datasets. Furthermore, sentences were not annotated with the corresponding dialect. In fact, we find that specifying the Arabic dialect is crucial as each Arabic dialect has unique vocabulary, grammar, and expressions. Ignoring these differences may lead to misinterpretation or misclassification of text. Moreover, some words or expressions can be neutral in one dialect but offensive in another. Besides, the language used in one dialect may have different contextual meanings in another. So understanding the specific dialect helps in adapting hate speech models to the such cultural variations.

For these reasons, in our proposed multi-dialectal dataset, we ensured balancing between Arabic dialects along with balancing between hate speech categories. Furthermore, we annotated each sentence with the corresponding dialect. This approach aims to address the limitations observed in previous datasets, promoting a more comprehensive understanding of Arabic hate speech across diverse linguistic and cultural contexts.

## 3 Corpus overview

In this section, we introduce ADHAR, which is a multi-dialectal and multi-category hate speech corpus for Arabic including 70,369 words and 4,240 tweets. We also report on the steps taken for data collection and annotation. Compared to existing corpora, our dataset stands out by covering multiple Arabic dialects and four categories of hate speech while maintaining high balance between the dialects and the hate speech categories.

Our dataset accommodates four primary regional dialects: Egyptian, Palestinian/Jordanian (Levant region), Khaleeji/Gulf (Gulf region), Maghrebi, along with Modern Standard Arabic (MSA) as the conventional and official communication Arabic language variant. Tweets included in our dataset belong to four categories: nationality, religious beliefs, ethnicity, and race. For each category, our corpus includes over 1,000 tweets that were collected from Twitter. These tweets are balanced across dialects so that our dataset includes at least 200 tweets for each combination of category and dialect. Furthermore, these 200 tweets are balanced so that our corpus includes for each category and for each dialect 100 tweets that have hate speech and 100 tweets that do not include hate speech. In total, our corpus includes 70,369 words and 4,240 tweets. Examples of tweets from our dataset are shown in Table 2. In the first example, the specific Egyptian word بعدين is used, which means then in English. In the second example, the specific Levantine word قديش is used, which means how or how much.

**Table 2:** Examples of hateful tweets from each category with different dialects.

**Category**	**Dialect**	**Sample tweet**	**Translation**
Nationality	Egyptian	ياجزائرييامعفنروحنظفبلدكمنالفسقوالرذيلةبعدينتكلمعنالمملكة	You dirty Algerian go clean your country from immorality and indecency then talk about the Kingdom
Religious beliefs	Jordanian/ Palestinian	ياربيقديشاليهودأغبياء	Oh God, how stupid Jews are
Ethnicity	Maghrebi	ليسوعرببلامازيغمانتشرفوشبجيرتهمنهائيا	They are not Arabs, but Berbers. We are not honored to be their neighbors at all
Race	Gulf	الاسيوينأشكالهمتقرفيخلونكتستفرغلمنتتفرجوصخينأكثرشيمقرفين	Asians are disgusting, they make you vomit when you see them, they are the dirtiest and the most disgusting

### 3.1 Data collection

After deciding on the four categories of tweets in our dataset (i.e., nationality, religious beliefs, ethnicity, race), we collected the data manually from Twitter using some seed keywords and some examples of these are shown in Table 3. We gathered a list of seed keywords for each dialect and for every category, which included dialectal hate words that are commonly used in the respective regions when describing people from certain nationality, religion, ethnicity, or race. On average, each dialect had 10–20 keywords in each category. Furthermore, we included general insults or slurs specific to each dialect, combining them with ordinary words associated with the four categories (see Appendix A). This approach enabled us to collect a high number of hateful tweets. We also integrated commonly used words exclusive to each dialect in daily conversations. These words conveyed basic meanings, including expressions like “What”, “Like this”, “I want”, “Seriously”, etc., enhancing our ability to focus on identifying tweets specifically written in the corresponding dialect. For example, as shown in Table 2, the word قديش, which means “how much” from the Jordanian/Palestinian dialect, and the word يخلونك which means “make you” from the Gulf dialect, are words that are only used in their respective dialect.

**Table 3:** Examples of tweets from each category with different dialects.

**Category**	**Dialect/language variant**	**Sample keywords**	**Translation**
Nationality	Maghrebi	بلدمليونونصفعبيط; الكرغولية; بلد سرقستان و كذوبستان; الخرائر; تريكة السنغول; سنغولي; الشعب الحمروكي; الزازايير	A country of a million and a half idiots^a^
Religious beliefs	MSA	دينإرهاب; العقيدة القذرة; مجوسي; كافر; الالحاد; كفرة;	The religion of terrorism; The dirty doctrine; Magian; Kafir; Atheism; Kafirs
Ethnicity	Egyptian	بربر;اسود; صعيدى; امازيغي	Berber; Black; Sa'idi; Amazighi
Race	Gulf	ابيض; اسود; افريقي; زنجي; عبيد; اسيوي	White; Black; African; Nigger; Slave; Asian
General hate	Jordanian/ Palestinian	كلب; حمار; متخلف; وسخ; ملعون; زبالة; همج	Dog; Donkey; Underdeveloped; Dirty; Cursed; Trashy; Barbarian

^a^The other Arabic expressions cannot be directly translated into English because they are offensive inventions, comprised of non-standard language, and derogatory terms, used to make fun of particular nationalities.

In order to be included in our dataset, each tweet had to meet the following requirements. First, it has to be written in Arabic with correct spelling and valid grammar, either in MSA or in an Arabic dialect. Second, the tweet has to be directly related to one of the categories. Third, the full tweet (i.e., multiple sentences can make up a tweet) must express a clear hatred/non-hatred toward a specific target depending on the category. To assess if a tweet includes hate speech or not we used the definition of Tontodimamma et al. (2021), which states that hate speech is any text that belittles an individual or a group based on attributes such as race, color, ethnicity, gender or religion.

### 3.2 Data balancing

**Figure 1:**
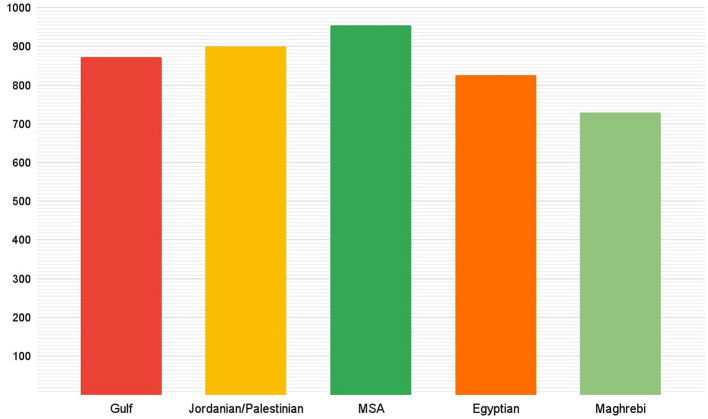
Number of tweets per Arabic variant in ADHAR.

One limitation of existing hate speech datasets for Arabic is their imbalance and we aimed at addressing this limitation in our dataset. To that end, we defined the following requirements when collecting the data. First, each of the four categories should have at least 1,000 tweets. Second, in each category, at least 500 tweets should include hate speech and at least 500 should not include hate speech. Third, in each dialect and in each category there should be at least 200 tweets divided between the two classes: hate and non hate. Statistics about these requirements and how our dataset fulfills them are given in Table 4. We established a target number of tweets to gather for each dialect and we met that target successfully. As some dialects had higher presence on Twitter, we were able to collect more tweets for them as shown in Figure 1.

**Table 4:** Number of tweets per class and category in ADHAR.

**Category**	**Hate**	**Not Hate**	**Total**
Nationality	583	508	1,090
Religious beliefs	514	536	1,050
Ethnicity	541	520	1,061
Race	513	522	1,035

**Figure 2:**
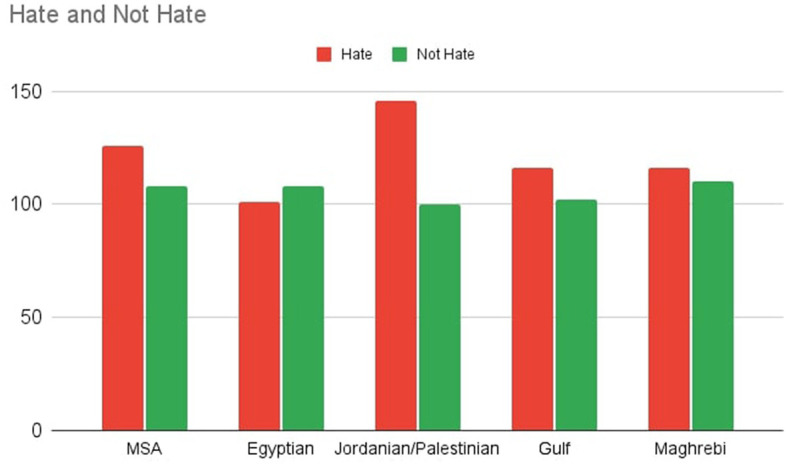
Distribution of nationality related tweets per Arabic variant.

As an example for the high level of balance in our dataset, Figure 2 shows the distribution of tweets per classes and dialects for one of the category nationality and the exact numbers are given in Table 5.

**Table 5:** Distribution of hate vs not hate tweets per Arabic variant for the category nationality.

	**Hate**	**Not Hate**	**Total**
MSA	122	103	225
Egyptian	100	103	203
Jordanian/Palestinian	144	100	244
Gulf	108	100	208
Maghrebi	108	102	210

To guarantee that the previously mentioned criteria are satisfied, we created charts for each category that tracks the data as it is input, as shown in Figure 2. Those charts track the overall number of tweets in each dialect, the number of tweets in each class, and the total number of tweets for the whole category. The exact numbers for each hate class per dialect are provided in Table 5.

The “Maghreb” category within our dataset includes a diverse range of Arabic dialects from the Maghreb region, including Algeria, Libya, Tunisia, and Morocco. This category comprises 840 entries, accounting for approximately 20% of the total dataset entries. The entries within this dialect group are characterized as follows: there are 430 entries marked as Hate Speech and 413 as Not Hate, indicating a relatively balanced distribution. Additionally, thematic categories exhibit equal distribution among the hate speech categories, with Ethnicity, Nationality, Religion, and Race each comprising approximately 210–216 entries (see Table 6). The Maghreb category also includes tweets that use a variant of Arabic that we refer to as generic Arabic. This variant is primarily based on MSA but does not follow its linguistic norms.

**Table 6:** Statistics for dialects within the “Maghrebi” dialect in ADHAR corpus across hate categories.

**Sub-dialect**	**Nationality**		**Religious beliefs**		**Race**		**Ethnicity**	
	**Hate**	**Not hate**	**Hate**	**Not hate**	**Hate**	**Not hate**	**Hate**	**Not hate**
Moroccan	38	37	41	45	33	30	44	46
Tunisian	3	20	5	31	20	35	7	33
Algerian	58	35	59	24	49	27	36	15
Libyan	7	6	3	4	2	8	6	5
Generic	2	4	2	2	1	3	22	8
Total	108	102	110	106	105	103	115	107

### 3.3 Data annotation

The annotations were done by three members of our research team who were native Arabic speakers and experts in multiple Arabic dialects. The annotation process went through three stages:

**First annotation:** For each dialect and each category, the tweets were annotated by the team member who collected them into two classes “Hate” and “Not Hate”. We made sure that the assigned annotator was an expert in the respective dialect, either being a native speaker of that dialect, or due to extensive exposure to that dialect.

**Second Annotation:** The annotators were assigned to annotate a specific dialect where the first annotation was done by a different annotator, while hiding the first annotation. We also ensured here that the second annotators had some good exposure to the respective dialect. In this stage the tweets of each category were classified into “Hate”, “Not Hate”, and “Discuss”.

**Final annotation:** A meeting involving all three members was held to discuss the cases where the first and the second annotations did not match or the cases where the tweets we classified as “discuss” by the second annotator. This stage resulted into either removing the tweets from the corpus if all involved annotators agreed on it being unclear, or changing the label of the tweet based on the agreement of all annotators, or moving the tweet to a different category because it fits better there.

In order to measure the quality of the annotation, we calculated the inter-annotator agreement using Cohen's Kappa as shown in Table 7. For all categories the values are above 0.9, which reflects a high level of agreement.

**Table 7:** Inter-annotator agreement using Cohen's Kappa.

**Hate speech category**	**Cohen's Kappa**
Nationality	0.9636
Religious beliefs	0.9605
Race/Ethnicity	0.9257
Overall	0.9489

## 4 Experiments

This section presents the methodology and results of our machine learning experiments, which we conducted to evaluate and test our dataset.

### 4.1 Setup

We conducted a set of machine learning experiments, in which the input to our models is Arabic text data, along with the corresponding dialect and the output is a binary classification: either “hate” or “non-hate”, along with the corresponding category, namely nationality, religious beliefs, ethnicity, or race. In fact, it is crucial to specify the dialect as part of the input because Arabic dialects can vary significantly, and a word that is considered harmful in one dialect might be interpreted as safe in another, due to cultural differences between Arab countries.

We divided our dataset into training and testing sets following a 75:25 ratio. Each instance in the dataset consists of a tweet, its corresponding dialect, the annotation class (i.e., hate or not hate), and the category (i.e., nationality, religious beliefs, ethnicity, race).

**Figure 3:**
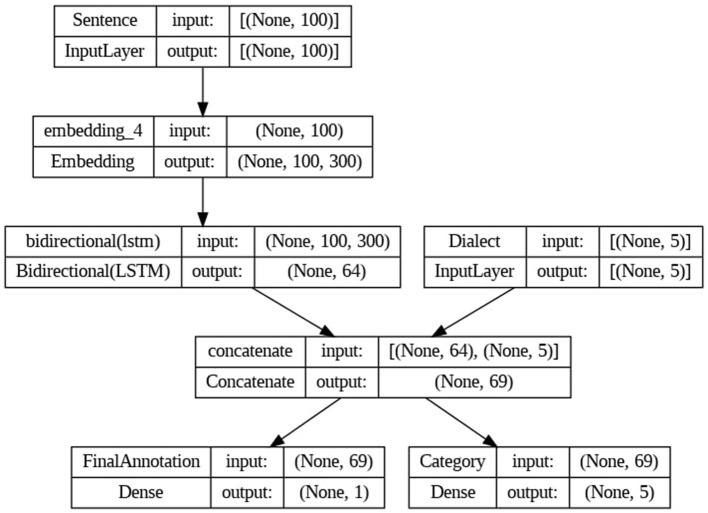
Architecture of CNN-BiLSTM model.

To pre-process the text data, we removed URLs, emails, stop words, punctuation, and non-Arabic characters. Then, we used a set of feature extraction techniques, including word n-grams, character n-grams, TF-IDF and word embeddings. These methods enabled us to extract relevant features from the text data. For word n-grams, we defined an n-gram range of (1,3), covering unigrams, bigrams, and trigrams. As for character n-grams, we set the range to (2,5). For character and word n-gram vectorizations, the representation of words is based on raw counts of each n-gram. This indicates the frequency of occurrence of each character or word n-gram in the text. For TF-IDF vectorization, the counted elements specifically correspond to words. Afterwards, we trained a range of machine learning models, including classical and neural network classifiers, to determine the best for our classification task. We started with classic machine learning classifiers like Support Vector Machines (SVM), Logistic Regression (LR), Random Forest, and Decision Trees. Following that, we examined the Multi-Layer Perceptron (MLP), a neural network model that uses many layers of nodes to learn input classification by altering inter-node weights.^3^ Additionally, we developed a Convolutional Neural Network - Bidirectional Long Short-Term Memory (CNN-BiLSTM) model, using its integrated convolutional and recurrent layers to conduct an in-depth analysis as shown in Figure 3. We explored various hyperparameter combinations to identify the optimal configuration.

All experiments were conducted using Google Colab,^4^ which served as the cloud-based computing infrastructure. It provided the essential computational resources required for training and evaluating the models. We also used the *pandas* package to edit and analyze the data., and the *nltk* package during the preprocessing for the tokenization and stop word removal tasks. Moreover, we used the *sklearn* package to train and evaluate all machine learning classifiers. For the implementation of the CNN-BiLSTM model, we used the *Keras* library, which is an open-source neural networks API written in Python, with a word embeddings dimension of 300.

As previously stated, the Arabic language includes various dialects, each with its own unique expressions and linguistic nuances. To investigate the impact of these dialectal differences on hate speech detection, we used the MLP model, which demonstrated the best performance with character n-gram feature extraction (as shown in Section 4.2). We trained this model on tweets written in one dialect and then tested its performance on tweets from the other studied dialects. Table 8 displays the accuracy scores for hate speech detection across different language variants examined in this study, including MSA, Egyptian, Gulf, Levantine (Jordanian/Palestinian), and Maghrebi (including Moroccan, Algerian, Tunisian, Libyan). The columns represent the language variants used for training, while the rows indicate those used for testing. Overall, the table shows noticeable variations in accuracy scores. The lowest accuracy of 0.64 was observed when training the model on tweets in the Egyptian dialect and testing it on Gulf tweets. In contrast, the highest accuracy score of 0.84 was achieved when the model was trained on Gulf dialect and then tested on Levantine dialect. This wide range and the fluctuations observed across these language variants underscore the importance of specifying the dialect during the annotation process in hate speech corpora for Arabic. It also emphasizes the importance of including different dialects alongside MSA in a balanced approach, rather than treating all Arabic sentences uniformly without regard to their specific dialects.

**Table 8:** Accuracy of hate speech detection across Arabic variants.

		**Training group**				
		**MSA**	**Gulf**	**Egyptian**	**Levantine**	**Maghrebi**
Testing group					
		MSA	-	0.70	0.69	0.66	0.76
		Gulf	0.72	-	0.64	0.82	0.74
		Egyptian	0.77	0.74	-	0.73	0.79
		Levantine	0.68	0.84	0.72	-	0.73
		Maghrebi	0.76	0.68	0.70	0.68	-

Furthermore, we used for our task the pre-trained language model AraBERT, specifically designed for Arabic text processing and built upon the BERT architecture. AraBERT, based on the Transformer architecture, provides comprehensive contextual understanding of Arabic language nuances and semantics. Its adaptability allows for fine-tuning on specific tasks, enabling us to adapt its representations to our hate speech detection task effectively.

### 4.2 Results

In our experiments, we initially chose to retain punctuation marks and stop words within the textual data, aiming to preserve contextual information and linguistic nuances. However, the performance was lower than when removing them, so we opted to omit them in order to achieve better results. For the BiLSTM model, we conducted various experiments with different hyperparameters. The configuration that produced the best performance is as follows: units = 32, dropout = 0.3, optimizer = Adam, batch size = 32, and learning rate = 0.001. To tune these hyperparameters, we used a separate validation dataset instead of the test data. This approach ensures the validity of the results by keeping the test data unseen during the hyperparameter tuning process. Table 9 shows the evaluation results for various classifiers using our dataset in the binary classification task for hate speech detection (the output is whether a tweet includes hate speech or not). These classifiers are evaluated with four distinct feature sets: TF-IDF, n-grams, and character-level features are used for Support Vector Machine, Logistic Regression, Random Forest, Decision Tree, and Multi-Layer Perceptron (MLP) while word embeddings are used for the CNN-BiLSTM model. Similarly, Table 10 shows the results we obtained for hate speech category detection.

**Table 9:** Results of different models for binary classification of (hate/not hate) on our corpus.

**Classifier**	**Features**		**Accuracy**	**Precision**	**Recall**	**F1-score**
	n-grams	Char	0.83	0.83	0.83	0.83
Support Vector Machine		Word	0.74	0.78	0.74	0.73
	TF-IDF		0.87	0.87	0.87	0.87
	n-grams	Char	0.88	0.88	0.88	0.88
Logistic Regression		Word	0.85	0.85	0.85	0.85
	TF-IDF		0.86	0.86	0.86	0.86
	n-grams	Char	0.80	0.80	0.80	0.80
Random Forest		Word	0.80	0.82	0.80	0.82
	TF-IDF		0.82	0.83	0.82	0.82
	n-grams	Char	-	-	-	-
Decision Tree		Word	0.79	0.79	0.79	0.79
	TF-IDF		0.78	0.78	0.78	0.78
	n-grams	Char	**0.90**	**0.90**	**0.90**	**0.90**
Multi Layer Perceptron		Word	0.88	0.88	0.88	0.88
	TF-IDF		0.88	0.88	0.88	0.88
CNN-BiLSTM	Word embeddings		0.85	0.82	0.89	0.85

The best performance values are indicated in bold.

**Table 10:** Results of different models for hate speech category classification on our corpus.

**Classifier**	**Features**		**Accuracy**	**Precision**	**Recall**	**F1-score**
	n-grams	Char	0.87	0.88	0.87	0.88
Support Vector Machine		Word	0.65	0.80	0.65	0.66
	TF-IDF		0.88	0.89	0.88	0.88
	n-grams	Char	0.91	0.91	0.91	0.91
Logistic Regression		Word	0.85	0.86	0.85	0.85
	TF-IDF		0.88	0.88	0.88	0.88
	n-grams	Char	0.90	0.90	0.90	0.90
Random Forest		Word	0.84	0.86	0.84	0.84
	TF-IDF		0.85	0.86	0.85	0.85
	n-grams	Char	0.76	0.76	0.76	0.76
Decision Tree		Word	0.83	0.84	0.83	0.83
	TF-IDF		0.81	0.81	0.81	0.81
	n-grams	Char	**0.92**	**0.92**	**0.92**	**0.92**
Multi Layer Perceptron		Word	0.89	0.89	0.89	0.89
	TF-IDF		0.88	0.89	0.88	0.88
CNN-BiLSTM	Word embeddings		0.86	0.86	0.86	0.86

The best performance values are indicated in bold.

Among the classifiers created using the different features extraction methods, the MLP classifier achieved the highest performance when using n-gram character features with an accuracy and F1-score of 90% for hate speech annotation, and an accuracy and F1-score of 92% for the hate speech category detection (i.e., nationality, religious beliefs, ethnicity, race), as shown in Tables 9, 10. However, using AraBERT resulted in superior performance, with accuracy and F1-scores reaching up to 94% for hate speech detection and up to 95% for category detection as shown in Tables 11, 12.

**Table 11:** Performance for hate speech detection using AraBERT (Hate/Not Hate).

**Classifier**	**Accuracy**	**Class**	**Precision**	**Recall**	**F1-score**
		Hate	0.96	0.93	0.94
AraBERT	**0.94**	Not Hate	0.92	0.95	0.94
				**Macro F1**	**0.94**

The best performance values are indicated in bold.

**Table 12:** Performance for hate speech category detection using AraBERT (Nationality/Religious beliefs/Ethnicity/Race).

**Classifier**	**Accuracy**	**Class**	**Precision**	**Recall**	**F1-score**
		Nationality	0.94	0.92	0.93
AraBERT	**0.95**	Religious beliefs	0.97	0.94	0.95
		Ethnicity	0.94	0.96	0.95
		Race	0.95	0.96	0.96
				**Macro F1**	**0.95**

The best performance values are indicated in bold.

## 5 Discussion

The ADHAR dataset introduced in this paper has several notable strengths that address crucial gaps in existing Arabic hate speech corpora. Its multi-dialectal nature, encompassing five major Arabic variants (Modern Standard Arabic, Egyptian, Levantine, Gulf, and Maghrebi), is a significant contribution. By incorporating multiple Arabic variants and annotating each sentence with its corresponding dialect, the dataset enables the development of more robust and culturally-aware hate speech detection models for Arabic. This approach acknowledges the linguistic variations across the Arab world and the potential for words or expressions to be interpreted differently depending on the dialect.

Another strength of ADHAR is its balanced distribution across dialects, hate/non-hate classes, and four key hate speech categories (nationality, religion, ethnicity, and race). Maintaining this balance is crucial for unbiased training and evaluation of machine learning models, as imbalanced datasets can lead to biased or skewed results. The rigorous data collection and annotation process, involving multiple native Arabic speaker annotators and measuring inter-annotator agreement, further enhances the quality and reliability of the dataset.

Furthermore, we carefully collected tweets within our dataset representing various Arabic dialects and language variants, ensuring a balanced distribution among them. This comprehensive approach was crucial due to the diverse nature of Arabic dialects. To confirm the importance of this methodology, we conducted an experiment where we trained our model on one language variant and evaluated its performance on another. This allowed us to observe fluctuations in accuracy across these language variants, highlighting the nuanced differences among dialects. These results underscore the significance of incorporating various dialects alongside MSA in a balanced approach, rather than collecting Arabic sentences without consideration for dialects, as seen in previous Arabic hate speech detection studies. It is worth noting that our dataset is the first to prioritize a balanced distribution among MSA and different Arabic dialects.

The experiments conducted using various classical and deep learning models demonstrate the effectiveness of ADHAR in developing accurate hate speech classifiers for Arabic. The best models achieved accuracy and F1-scores of up to 94% for hate speech detection and up to 95% for category detection when using the pre-trained AraBERT model. These promising results highlight the potential of ADHAR in driving future research and applications in Arabic NLP for hate speech detection.

On the other hand, our dataset and study also have some limitations. While the dataset covers four language variants, it does not include all Arabic dialects. Expanding the dataset to incorporate a wider range of dialects would further enhance its representativeness and applicability across the diverse Arabic-speaking regions.

A further potential limitation is the static nature of the dataset. As language evolves, and new hate speech patterns or expressions emerge, the dataset may require periodic updates to remain relevant and comprehensive. Establishing a mechanism for continuous data collection and annotation could help address this limitation. Another limitation of our work is that our study does not fully explore the generalizability of our models across different contexts and/or datasets.

Despite these limitations, the ADHAR dataset represents a significant contribution to the field of Arabic NLP, particularly in the domain of hate speech detection. Its multi-dialectal, multi-category, and balanced nature address crucial gaps in existing resources, paving the way for more robust and inclusive hate speech detection models for Arabic.

## 6 Conclusion

We presented in this paper ADHAR, a novel multi-dialect, multi-category hate speech dataset for Arabic with over 70,000 words. This dataset fills an important gap and provides a valuable resource for the research community. It includes hate speech related to nationality, religious beliefs, ethnicity, and race from around the Arab World. Manually annotated Arabic hate speech datasets that are well balanced are extremely rare, especially ones that cover multiple dialects and hate speech categories.

ADHAR is carefully balance across five major Arabic variants (Egyptian, Levantine, Gulf, Modern Standard Arabic, and Maghreb), across the two classes (hate and non hate) and across four key hate speech categories (nationality, religion, ethnicity, race). Maintaining this balance facilitates unbiased training and evaluation of machine learning hate speech classifiers, which is a significant advantage over previous imbalanced datasets. Our conducted machine learning experiments confirm this statement.

The ADHAR dataset and the hate speech detection models developed using it have significant potential for real-world applications and positive societal impact. One major application would be to integrate these models into content moderation systems for social media platforms, forums, and other online communities in the Arab world. By automatically detecting hate speech across different Arabic dialects and categories like nationality, religion, ethnicity and race, such systems could help create safer online spaces by filtering out abusive and hateful content before it spreads. This could reduce harm caused by hate speech, promote more respectful online discourse, and make Internet communities more inclusive. Beyond social media, these models could also be applied in education to detect cyberbullying among students or in workplace settings to prevent harassment and discrimination. Additionally, the insights from analyzing patterns of hate speech could inform initiatives to raise awareness, counter hate rhetoric, and encourage tolerance in the Arabic-speaking regions.

While ADHAR represents a significant step forward in creating a comprehensive multi-dialectal hate speech corpus for Arabic, there is still room for further expansion and improvement. One promising direction for future work is to broaden the dataset's coverage of Arabic variants beyond the five currently included (Egyptian, Levantine, Gulf, Modern Standard Arabic, and Maghrebi). For instance, the corpus can be extended to cover such as the Iraqi dialect.

Furthermore, future work could explore the linguistic and cultural nuances that influence hate speech expressions across different Arabic dialects. While the current study acknowledges the potential for words or expressions to have varying meanings or connotations depending on the dialect, a more in-depth analysis of such instances could yield valuable insights. This understanding could inform the development of more context-aware and culturally-sensitive hate speech detection models for Arabic. A third direction of future work could study the generalizability of our models across different contexts and/or datasets.

By expanding ADHAR to encompass a wider range of Arabic dialects and delving deeper into the linguistic and cultural factors that shape hate speech, future research can build upon the foundations laid by this study. This would contribute to the development of more comprehensive and inclusive hate speech detection systems, capable of addressing the diverse linguistic landscape of the Arabic-speaking world.

## Author contributions

AC: Methodology, Supervision, Validation, Writing – review & editing. MB: Data curation, Methodology, Writing – original draft. RA: Data curation, Methodology, Writing – original draft. AA: Data curation, Methodology, Writing – original draft. WZ: Funding acquisition, Project administration, Writing – review & editing.

## Footnotes

^1^ https://edinburghnlp.inf.ed.ac.uk/workshops/OSACT4/

^2^ https://osact-lrec.github.io/

^3^These models were trained using the default settings provided by the sklearn library.

^4^ https://colab.research.google.com/
