# Supplementary material for: Hate speech detection with ADHAR: a multi-dialectal hate speech corpus in Arabic
Source: Front Artif Intell. 2024 May 30;7:1391472. doi: 10.3389/frai.2024.1391472 (PMC11170444; doi:10.3389/frai.2024.1391472)
Supplement: Supplementary file 1 [file Data_Sheet_1.pdf]

SEED KEYWORDS FOR DATA COLLECTION

For the nationality category, the keywords included:

لاجئين، المصريين، المصاروة، الفراعنة، فلسطيزي، الفلسطن، الفلسطينين، الفسطيزيين، البلاجكة، الاردنة، الاردنين، ابو فلس، السوريين، السوارنة، الفينيئين، السعاودة، المراركة، العراكيين، الالمان، الشعب الحمروكي، كرغولي ، احفاد ابو لهب، السناغلة ، البوسبيرين، الكرغولية، بلاد المليون و نصف شهيد، بلد مليون و نصف عبيط ، بلد سرقة و كذب، خاوه خاوه، طاوه طاوه، سنغولي، مروكي بوسبيري، [illegible] زايرخة، زائير، خرة [illegible]

[illegible] البخاري [illegible] التوانسا، وانا المغاربة، كرغل ،

Translation :
Refugees, Egyptians, Al-Masarwa (Referring offensively to Egyptians), Pharaohs, Philastizi (Written offensively referring to Palestinians), Al-Phalastan (Written offensively referring to Palestinians), The Palestinians, Al-Phalastizin (Written offensively referring to Palestinians), Belgians (Referring offensively to Palestinians), The Jordanians (Written offensively), Jordanians, Abu Falas (Referring offensively to Palestinians), Syrians, Al-Sawarna (Referring offensively to Syrians), Phoenicians, Al-Saawda (Referring offensively to Saudis), Moroccans, Al-Irakyeen (Referring offensively to Iraqis), Germans, Hamrouk people (Referring offensively to Moroccans), Kargoli (Referring offensively to Algerians), Abu Lahab's grandchildren, Senegaleses (Referring offensively to Moroccans), Bosperyeen (Referring offensively to Moroccans), Al-Kargolyah (Referring offensively to Algerians), The country of a million and a half martyrs (Referring to Algeria), A country of a million and a half idiots (Referring offensively to Algerians), The country of Robbery and Lying (Referring offensively to Algerians), Khawa Khawa (Referring to Algerians), Tawa Tawa (Referring offensively to Algerians), Sangoli (Referring offensively to Moroccans), Maroki Bospery (Referring offensively to Moroccans), The offspring of the Senegalese (Referring offensively to Moroccans), Moroccans, Sons of Bosper (Referring offensively to Moroccans), Moroccans, Kharair (Referring offensively to Algeria), Algeria, Algeria, Our Tunisian brothers, Our Moroccan brothers, Kargal (Referring offensively to Algerians), The Libyans, Qataris, Saudis, Tunisians, The Turks, Al-Daziria (Referring to Algerians), Slaves of Al-Bukhari.

For the religious beliefs category, the keywords included:

المسلمين، مسلم ، مسلمة ، المسيحيين، مسيحي، مسيحية ، اليهود ، يهودي، يهودية ، كفتس ، وهابي ، سلفي ، عبد تواضروس، عبد شنودة ، إسلامجي ، كافر ، أعداء الإسلام، إرهاب، العقيدة القذرة ، الشيعة ، سني ، كفرة ، الالحاد ، يزيديين، علويين، مجوسي، ملحد ، ماروني، نصراني، علماني، الإسلام دين إرهاب، رجعيين، دواعش، الشيعه المجوس، روافض ، الرسول، الإسلام، الاديان، صلاة ، النبي ، الدين ، الديانة .

Translation:
Muslims, Male Muslim, Female Muslim, Christians, Male Christian, Female Christian, Jews, Male Jewish, Female Jewish, Kaftas (Referring offensively to Christians), Wahabi, Salafi, Salves of Tawadros (Referring offensively to Christians), Slaves of Shenouda (Referring offensively to Christians), Islamji (Referring offensively to Muslims), Infidel, Enemies of Islam, Terrorism, Dirty Doctrine, Shia, Sunni, Infidels, Atheism, Yazidis, Alawites, Magian, Atheist, Maronite, Christian, Secular, Islam the religion of terrorism, Reactionaries, ISIS, Shiite Magians, The shiite, The Messenger, Islam, Religions, Prayers, the

Prophet , The Religion , Religion.

For the ethnicity category, the keywords included:

خليجي، صعيدى، البدو، العرب، الفرس، الامازيغ، البربر، كنعانيين، الحضر، حضري، الشوام، الاكراد، الأمازيغ مجهولي النسب و الهوية، أمة الزيغ، اخرابي، بربر همج، حثاله الامازيخ، بدو، البربري الافريقي، بربر، بول بعير، جلف الصحراء، أعرابي، أعرابية، البنغال البدو، الاماطيز، الاماعيز، المستعرب، عربوجي، عربوجية، البربريست، خرافة الأمازيغ، البدو الهمج، احفاد ابو لهب، قريش، كلب البنغال، قومية البدو، مخلفات البنغال، نسل البنغال و الهنود، الاماسيخ ، القبائلية، الشاوية، الميزابية، الشلوح.

Translation:
Khaliji (Referring to Gulf people), Saaidi (Referring to ethnic people from southern Egypt), The Bedouins, Arabs, Persians, Amazighi people, The Berbers, Canaanites, Urban Arabs, Urban Arab, Al-Shawam (Referring to Levant people), Kurds, Amazigh of unknown lineage and identity, The people of deviation (Referring offensively to Amazighs), Akhrabi (Referring offensively to Arab people), Barbarians Savages (Referring offensively to Amazighs), Amazigh scum, Bedouin, African barbarian, Berber, Camel urine (Referring offensively to Arabs), Desert's boorish people (Referring offensively to Arabs), Arabic man, Arabic woman, Bengal Bedouins (Referring offensively to Arabs), Al-Amatyz (Referring offensively to Amazigh people), Al-Amayz (Referring offensively to Amazigh people), Al-Mostarib (Referring to people with non-Arab origins but live in an Arab environment), Araboji (Referring offensively to Arabs), Arabojiyah (Referring offensively to Arabs), barbarist (Referring offensively to Amazigh people), The myth of the amazigh, Savage Bedouins, Abu Lahab's grandchildren (Referring offensively to Arabs), Quraysh (Referring offensively to Arabs), Bengals' Dog (Referring offensively to Arabs), Bedouin nationalism, Bengal waste (Referring offensively to Arabs), Descendants of Bengals and Indians (Referring offensively to Arabs), Amazikh (Referring offensively to Amazigh people), Tribalism, Chaoui people, Mozabite people, Shilha people.

For the race category, the keywords included:

زنجي، اسيوي، ابيض، افارقة، افريقي، اسود، زنوج، السود، الهنود، الاسيوين، اوروبي، زنجي خادم، كحلوش، وصيف، وصفان، كحالش، اصحاب البشرة السوداء، عبيد البوخاري، عبيد البخاري، ماما أفريكا، افريقيا السوداء، عبد حبشي، محروق ، عنصري ، راسيست، الرجل الابيض.

Translation:
Nigger, Asian, White, Africans, African, Black, Niggers, Black people, Indians, Asians, European, Nigger servant, Kahloush (Referring to Black People), Wasif (Referring offensively to Black People), Wisfan (Referring offensively to Black People), Kahalish (Referring to Black People), People of black skin, Mama Africa, Black Africa, Abyssinian slave, Burned (Referring offensively to Black People), Racist , Racist, White man.

Additionally, we included general insult and slur words specific to each language variant:

- MSA:
  خونة، اغبياء، عديمين اخلاق، انذال، رجعيين، اسوء، اقذر، احقر، حقود.
  Translation: Traitors, Stupid, Immoral people, Scoundrel, Reactionaries, Worse, Dirtiest, The most despicable, Venomous.

- Egyptian dialect:
  معفن، غبي، متخلف، حمار، جربوع، حيوان، حقير، زبالة، كلب، واطي.
  Translation: Rotten, Stupid, A backward person, Donkey, Jarbou' (Insult in Egyptian dialect), Animal, Despicable, Garbage, Dog, lowlife.

- Jordanian/Palestinian dialect:
  كلب، حمار، واطي، منحط، عرص، متخلف، وسخ، ملعون، منيك، زبالة، همج، معفن.
  Translation: Dog, Donkey, lowlife, Decadent, Pimp, Backward, Dirty, Cursed, Manyak (Insult in Levantine dialect), Garbage, Savage, Filthy.

- Maghrebi dialect:
  مسخ، مسخين، أنذال، جربوع، جرابع، بغل، بغال، بهايم، بهيم، ماسيخ، الأوباش.
  Translation: Filthy, Dirty people, Scoundrel, Jarbou' (Insult in Maghrebi dialect), Jarabii' (Insult in Maghrebi dialect), Mule, Mules, Donkeys, Donkey, Filthy, Bastards.
